# Science and fiction in critical care: established concepts with or without evidence?

**DOI:** 10.1186/s13054-019-2419-4

**Published:** 2019-06-14

**Authors:** Martin Westphal

**Affiliations:** 1 Department of Anaesthesiology, Intensive Care and Pain Medicine, University of Muenster, Albert-Schweitzer-Campus 1, Building A1, 48149 Muenster, Germany; 2 Fresenius Kabi AG, Else-Kröner-Str. 1, 61352 Bad Homburg, Germany

**Keywords:** Evidence based medicine, Intensive care medicine, Critical care medicine, Oliguria, Crush syndrome, Kidney transplantation, Enteral nutrition, Mechanical ventilation, Sedation

## Abstract

In the absence of evidence, therapies are often based on intuition, belief, common sense or gut feeling. Over the years, some treatment strategies may become dogmas that are eventually considered as state-of-the-art and not questioned any longer. This might be a reason why there are many examples of “strange” treatments in medical history that have been applied in the absence of evidence and later abandoned for good reasons.

In this article, five dogmas relevant to critical care medicine are discussed and reviewed in the light of the available evidence. Dogma #1 relates to the treatment of oliguria with fluids, diuretics, and vasopressors. In this context, it should be considered that oliguria is a symptom rather than a disease. Thus, once hypovolaemia can be excluded as the underlying reason, there is no justification for giving fluids, which may do more harm than good in euvolaemic or hypervolaemic patients. Similarly, there is no solid evidence for forcing diuresis by administering vasopressors and loop diuretics. Dogma #2 addresses the treatment of crush syndrome patients with aggressive fluid therapy using NaCl 0.9%. In fact, this treatment may aggravate renal injury by iatrogenic metabolic acidosis and subsequent renal hypoperfusion. Dogma #3 concerns the administration of NaCl 0.9% to patients undergoing kidney transplantation. Since these patients are usually characterised by hyperkalaemia, the potassium-free solution NaCl 0.9%, containing exclusively 154 mmol/l of sodium and chloride ions each, is often considered as the fluid of choice. However, large volumes of chloride-rich solutions cause hyperchloraemic acidosis in a dose-dependent manner and induce a potassium shift to the extracellular space, thereby increasing serum potassium levels. Thus, balanced electrolyte solutions are to be preferred in this setting. Dogma #4 relates to the fact that enteral nutrition is often withheld for patients with high residual gastric volume due to the theoretical risk of gastro-oesophageal reflux, potentially resulting in aspiration pneumonitis. Despite controversial discussions, there is no clinical data supporting that residual gastric volume should be generally measured, especially not in patients without a gastro-intestinal surgery and/or motility disorders. Clinical evidence rather suggests that abandoning residual gastric volume monitoring does not increase the incidence of pneumonia, but may benefit patients by facilitating adequate enteral feeding. Finally, dogma #5 is about sedating all mechanically ventilated patients because “fighting” against the respirator may cause insufficient ventilation. This concern needs to be balanced against the unwanted consequences of sedation, such as prolonged mechanical ventilation and intensive care unit length of stay as well as increased risk of delirium. Modern concepts based on adequate analgesia and moderate to no sedation appear to be more suitable.

In conclusion, dogmas are still common in clinical practice. Since science rather than fiction should govern our actions in intensive care medicine, it is important to remain critical and challenge long established concepts, especially when the underlying evidence is weak or non-existing.

## Background

Medical history tells of many, once established therapeutic strategies and dogmas, which have been performed for decades despite the lack of solid evidence, and later abandoned without any further ado. Examples of such dogmas are the treatment of tuberculosis by inducing a “therapeutic” pneumothorax, mercury to heal syphilis, and lobotomy to cure a variety of mental disorders. While it is easy to dismiss these outdated dogmas from today’s “enlightened” perspective, we should not forget that such treatments were common practice well into the twentieth century. In fact, António Egas Moniz received the Nobel Prize for his discovery of the therapeutic value of lobotomy in certain psychoses in the year 1949. Therefore, it is time to ask ourselves, if we really know and do better at present, or if junior physicians of today may look back in dismay at some of our treatments, just as we look back at lobotomy today.

To better understand the implications of dogmas in medicine, first the definition of the term should be examined. “A dogma is a belief or set of beliefs that people are expected to accept without asking questions about them” (https://www.macmillandictionary.com/dictionary/british/dogma). Challenging them may result in social sanctions by their peer group. In medicine, this applies to common treatments whose rationale is derived from physiological considerations, gut feeling, common sense, assumptions, instinct, or attitudes, which have been little or never tested in rigorous clinical trials. This is why individual therapeutic strategies may become dogmas over time and are consequently considered as standard. The objective of this article is to challenge some of the current dogmas in intensive care medicine by scrutinising their scientific justification.

### Dogma #1: Give fluids, diuretics, and vasopressors if urinary output decreases

Most oliguric intensive care unit (ICU) patients receive fluids, diuretics, and/or vasopressors with the goal to prevent acute kidney injury (AKI). This treatment, however, is based on the belief that AKI in critically ill patients results from renal ischaemia. Whereas fluids and vasopressors are usually administered to increase renal blood flow and in proportion oxygen delivery, diuretics are given to reduce the osmolarity in the renal medulla. This is expected to increase glomerular filtration rate (GFR) and ultimately improve renal function [1].

Oliguria, however, is a clinical symptom rather than a disease. The underlying mechanisms include, but are not restricted to, hypovolaemia, physiologic stress response, tubular damage, post-renal obstruction or a combination of these factors. If hypovolaemia is not the cause of oliguria, it does not make sense to administer fluids for its treatment. Giving fluids to patients who are not hypovolaemic may in fact harm the kidneys by increasing intracapsular pressure, thereby further reducing urinary output. Hence, administering fluids to these patients may lead into a vicious cycle of infusing more and more fluids to treat oliguria, which is eventually a result of over-hydration [1]. Furthermore, if functional renal damage is present, e.g., due to ischaemia or nephrotoxins, treating oliguria with fluids or diuretics is unlikely to improve renal function. In this context, it is also noteworthy that the *Kidney Disease: Improving Global Outcomes* (KDIGO) guidelines for AKI recommend neither fluid therapy beyond the correction of hypovolaemia nor diuretics for the treatment of AKI [2]. This is supported by the fact that clinical evidence for a sustained increase in urine output or improvement in renal blood flow secondary to fluids and/or diuretics is lacking. Conversely, indiscriminately giving fluids to oliguric patients increases the risk of fluid overload with negative consequences on morbidity [3]. Although fluid overload has been identified as independent risk factor for AKI in critically ill patients [4], “fill and spill” (“fill” the circulation and urine will “spill”) is still a dogma in many ICUs [1]. Likewise, the concept of “squeeze and diurese” is commonly applied. It aims to increase mean arterial blood pressure with vasopressors (“squeeze”) and at the same time administer loop diuretics to paralyse the medulla and avoid ischaemia (“diurese”). Interestingly, no randomised controlled trials (RCTs) support the latter treatment strategies [1].

Taken together, there is no reliable evidence that administration of fluids, beyond the correction of hypovolaemia, results in a sustained increase in renal blood flow or renal oxygen delivery. On the contrary, solid clinical evidence shows that a positive fluid balance has negative consequences on clinical outcomes [1].

### Dogma #2: Treatment of crush syndrome with aggressive fluid resuscitation

Crush syndrome may result from trauma associated with massive muscular compression, rhabdomyolysis, and reperfusion injury. In the presence of rhabdomyolysis, the necrotizing muscle fibres release myoglobin, creatine phosphokinase (CK), lactate dehydrogenase (LDH) and intracellular electrolytes. When compression is released, reperfusion releases these substances into the systemic circulation, thereby causing electrolyte and metabolic imbalance, such as hyperkalaemia and metabolic acidosis. The most severe complications of crush syndrome include AKI, arrhythmia and liver injury. Though in this context the pathophysiology of AKI is not completely understood, it appears rational that the proteins released during rhabdomyolysis cause glomerular and tubular obstruction [5].

Currently, it is believed that aggressive fluid therapy, often combined with diuretics (forced diuresis) or hyperosmotic solutions like mannitol, dilutes unappreciated molecules and flushes them out of the kidneys. “Aggressive” denotes the intravenous administration of a combination of fluids, e.g. 1 part each of NaCl 0.9% and glucose 5%, and 100 mmol hydrogencarbonat per 2 l of volume. In large-scale emergencies, where close medical supervision of the individual patients is not possible, at least 3–6 l per day are recommended, while 10 l or more per day are common practice if continuous supervision from the moment of trauma rescue until discharge from the ICU is possible [6]. However, it is noteworthy that the evidence to support this approach is sparse and derived solely from animal trials and case reports.

In this context, it should be considered that the consequence of administering such large amounts of fluids is a positive fluid balance, which in turn is associated with risks in its own, e.g. pulmonary oedema and abdominal compartment syndrome, particularly if renal function declines during administration [4]. Given that aggressive fluid resuscitation with NaCl 0.9% may foster renal hypoperfusion and aggravate renal injury [7], the benefit/risk ratio should be carefully evaluated in each individual case. This seems to be especially important, since a strong association of hyperchloraemia with negative clinical outcomes including increased mortality has been demonstrated in many clinical settings including non-cardiac surgery [8].

Likewise, a chloride-restricted approach was associated with a better outcome including less requirement of new renal replacement therapy (RRT) and less persistent renal dysfunction versus NaCl 0.9% in a large pragmatic study of more than 15.000 critically ill patients [9]. Based on the published data, aggressive fluid therapy for crush syndrome requires very careful weighting of perceived benefits and potential risks of large volume resuscitation, especially when liberal concentrations of chloride are administered.

### Dogma #3: Patients undergoing kidney transplantation should receive NaCl 0.9%

As patients with chronic kidney disease (CKD) scheduled for kidney transplantation usually suffer from hyperkalaemia, interventions that further increase potassium plasma levels may theoretically increase the risk of serious cardiac adverse events. Since NaCl 0.9% does not contain potassium, it is often considered the solution of choice for patients undergoing renal transplantation. However, this reasoning does not consider the effect of NaCl 0.9% on the acid-base homeostasis. In this regard, it should be noted that large volumes of NaCl 0.9% may induce hyperchloraemic acidosis with a subsequent shift of potassium from the intracellular to the extracellular space due to a depletion of the physiological buffer mechanisms. Thus, NaCl 0.9% infusions are not innocuous for potassium plasma levels. In fact, substantial intravenous volumes of NaCl 0.9% cause an increase in potassium blood levels, which is just the opposite of what was intended by choosing NaCl 0.9% for renal transplant patients [10].

The first clinical trial specifically investigating the effects of NaCl 0.9% in renal transplantation randomised patients to receive either NaCl 0.9% or Ringer’s lactate for intraoperative intravenous fluid therapy. When an interim analysis of 51 patients showed that significantly more patients in the NaCl 0.9% group developed hyperchloraemic acidosis and hyperkalaemia, the trial was terminated prematurely for safety reasons [11]. These findings have been confirmed in a recent meta-analysis of four studies including 237 patients, which found significantly elevated postoperative potassium levels in patients receiving NaCl 0.9% for intraoperative fluid therapy [12]. Accordingly, it can be concluded that NaCl 0.9% is not a “physiological” solution and not the right choice for renal transplant patients.

### Dogma #4: Enteral nutrition (EN) is contraindicated in patients with a high gastric residual volume

It is a common belief that high residual gastric volumes (RGV) increase the risk of gastro-oesophageal reflux, thereby resulting in an increased risk of aspiration and pneumonia [13]. However, the available data supporting this assumption is sparse. In a study by McClave and colleagues [14], it was not possible to identify a threshold RGV level indicative for an increased risk of aspiration. In addition, the authors found no correlation between the incidence of pneumonia and the frequency of regurgitation or aspiration, which may be caused by high RGV. Hence, the study results do not support the use of RGV as a risk marker to guide administration of EN.

A more recent study investigated the effect of omitting routine RGV monitoring on the incidence of ventilator-associated pneumonia. The authors reported that abandoning routine RGV monitoring was not inferior to routine RGV monitoring regarding the incidence of ventilator-associated pneumonia [15]. Notably, the proportion of patients receiving 100% of their calculated caloric goal was significantly higher in the group without RGV monitoring (odds ratio; 1.77, 90% CI 1.25–2.51; *p* = 0.008), because enteral feeding was interrupted less frequently than in those with RGV measurements.

In conclusion, solid evidence for the benefits of general RGV monitoring is lacking. Although it might be meaningful in selected patients with gastro-intestinal surgery and perturbed motility, the consequences of withholding adequate enteral feeding should be taken into account for the overall risk assessment. In this context, Marik [13] argues that early EN is feasible for most ICU patients and improves clinical outcomes. Since early EN initiation is an indicator of the quality of care delivered in ICUs, it should be considered the standard of care. Thus, the decision of measuring RGV should be made individually (in selected high-risk patients) rather than being applied routinely.

### Dogma #5: All mechanically ventilated patients in the ICU require sedatives

The dogma that all patients with endotracheal tube and/or mechanical ventilation should receive intravenous sedation to improve their tolerance to mechanical ventilation is based on the belief that a patient “fighting” against the respirator will receive insufficient ventilatory support. However, this assumption needs to be balanced against the evidence that administration of sedatives to ICU patients has unwanted consequences. In the short term, sedation may cause respiratory depression, haemodynamic instability or metabolic acidosis. In the long term, sedation may prolong duration of mechanical ventilation and consequently ICU length of stay. Last but not the least, the increased risk of triggering acute delirium has to be taken into consideration [16]. To avoid the negative side effects of sedation, ICU patients receiving mechanical ventilation should be managed primarily by multimodal analgesia with little, if any, sedative medication. To that end, Vincent and colleagues developed the early comfort concept, relying on adequate analgesia, minimal sedation and maximum humane care (eCASH) [17]. With this approach, the first priority is effective pain management with flexible multimodal analgesia and minimal opioid use. Sedation is applied only on demand after adequate pain relief has been achieved.

## Conclusions

In conclusion, dogmas are still common in today’s clinical practice and, by no means, are restricted to critical care medicine. Since absence of evidence is not automatically evidence of absence [18], a dogma per se is not questionable. However, we should focus on the implementation of evidence-based knowledge in daily clinical practice and be open minded to adapt our strategies once new evidence that is not in harmony with a previous belief becomes available. While expert opinions are essential in the absence of evidence and in tailoring individual therapies, these opinions must stand the test of challenging colleagues. If the answer to the question “Why did you apply this specific therapy to this patient?” is simply and exclusively “Because we always did it this way”, then it might be time to take a second look at the current evidence. In this regard, the original definition of evidence-based medicine by David Sacket and colleagues should be considered: “It’s about integrating individual clinical expertise and the best external evidence” [19]. Accordingly, RCTs and meta-analyses are important, but not the one and only truth. Similarly, individual experience is crucial, but the experience of the “eminence” should not substitute the external evidence [20].

As Sir Arthur Conan Doyle famously stated: “Once you eliminate the impossible, whatever remains, no matter how improbable, must be the truth.” This implies that we should be (self-)critical and open minded to ensure that science, rather than fiction, governs our actions in critical care.

## Abbreviations

AKI: Acute kidney injury
CK: Creatine phosphokinase
CKD: Chronic kidney disease
eCASH: Early comfort using analgesia, minimal sedatives and maximal humane care
eGFR: Estimated glomerular filtration rate
EN: Enteral nutrition
GFR: Glomerular filtration rate
ICU: Intensive care unit
KDIGO: Kidney Disease: Improving Global Outcomes
LDH: Lactate dehydrogenase
RCT: Randomised controlled trials
RGV: Residual gastric volumes
RRT: Renal replacement therapy
